# Long COVID and hypermobility spectrum disorders have shared pathophysiology

**DOI:** 10.3389/fneur.2024.1455498

**Published:** 2024-09-05

**Authors:** Ravindra Ganesh, Bala Munipalli

**Affiliations:** ^1^Division of General Internal Medicine, Mayo Clinic, Rochester, MN, United States; ^2^Division of General Internal Medicine, Mayo Clinic, Jacksonville, FL, United States

**Keywords:** long COVID, hypermobile Ehlers–Danlos syndrome, hypermobility spectrum disorders, myalgic encephalomyelitis/chronic fatigue syndrome, postural orthostatic tachycardia syndrome

## Abstract

Hypermobility spectrum disorders (HSD) and hypermobile Ehlers–Danlos syndrome (hEDS) are the most common joint hypermobility conditions encountered by physicians, with hypermobile and classical EDS accounting for >90% of all cases. Hypermobility has been detected in up to 30–57% of patients with myalgic encephalomyelitis/chronic fatigue syndrome (ME/CFS), fibromyalgia, postural orthostatic tachycardia syndrome (POTS), and long COVID (LC) compared to the general population. Extrapulmonary symptoms, including musculoskeletal pain, dysautonomia disorders, cognitive disorders, and fatigue, are seen in both LC and HSD. Additionally, ME/CFS has overlapping symptoms with those seen in HSD. Mast cell activation and degranulation occurring in both LC and ME/CFS may result in hyperinflammation and damage to connective tissue in these patients, thereby inducing hypermobility. Persistent inflammation may result in the development or worsening of HSD. Hence, screening for hypermobility and other related conditions including fibromyalgia, POTS, ME/CFS, chronic pain conditions, joint pain, and myalgia is essential for individuals experiencing LC. Pharmacological treatments should be symptom-focused and geared to a patient’s presentation. Paced exercise, massage, yoga, and meditation may also provide benefits.

## Introduction

Hypermobility spectrum disorders and hypermobile Ehlers–Danlos syndrome (EDS) are the most common joint hypermobility conditions encountered by physicians (1). In 2017, the International Classification of Ehlers–Danlos syndromes was introduced to replace previous terms used to describe symptomatic joint hypermobility and outline appropriate diagnostic criteria (1–3). Under the new classification, the term hypermobile EDS (hEDS) was introduced to replace the previous terms, and the term hypermobility spectrum disorders (HSD) was introduced to represent patients who do not meet the diagnostic criteria for hypermobile EDS (1). The diagnostic criteria for hEDS and HSD are designed to exclude other rare conditions that may present with joint hypermobility, such as other types of EDS and heritable connective tissue disorders.

The prevalence of hypermobile EDS is approximately 1 in 5,000 births, and in conjunction with classical EDS, it accounts for 90% or more of all cases of EDS (1, 2, 4). Similarly, the prevalence of HSD is estimated to be approximately 10–20%, with a higher prevalence in children and adolescents (5). Hypermobility disorders manifest more frequently in women, similar to the sex prevalence seen in other complex chronic illnesses including myalgic encephalomyelitis/chronic fatigue syndrome (ME/CFS), fibromyalgia, postural orthostatic tachycardia syndrome (POTS), and most recently, long COVID (LC) (6–8). Joint hypermobility has been detected in up to 57% of patients with POTS, 49% of patients with ME/CFS, 30% of patients with LC, and 27% of patients with fibromyalgia, compared to 10–20% in the general population (5, 9–12).

While the inheritance pattern of hEDS is autosomal dominant, there is no clear genetic etiology that has been identified, though recent data have provided some candidate mutations, including the *MIA3* gene encoding a transport protein essential in collagen synthesis, which was abnormal in 13/100 patients with HDS in one series, and the *ELN* gene encoding for elastin (13, 14).

Clinical features of hEDS include joint hypermobility, skin findings, joint pains, and recurrent dislocations. Hypermobile EDS and, to a lesser extent, HSD may also be associated with several extra-articular symptoms, including anxiety disorders, chronic pain, fatigue, orthostatic intolerance, functional gastrointestinal disorders, and pelvic and bladder dysfunction (1). In addition to musculoskeletal complaints, non-musculoskeletal complaints are common in patients with hEDS/HSD, with dysautonomia being identified on autonomic function tests in many patients (3). Symptoms of dysautonomia are related to lower quality of life, physical impairment, fatigue, and affective distress, with a similar autonomic symptom burden to fibromyalgia (15). Autonomic dysfunction is characterized by sympathetic overactivity at rest with impaired sympathetic response to acute stressors, with excessive sympathetic tone leading to pain amplification (15).

## Relationship to post-infectious syndromes including long COVID

The risk of developing persistent symptomatology after infection with severe acute respiratory syndrome coronavirus 2 (SARS-CoV-2), also known as post-acute sequelae of COVID-19 (PASC) or long COVID (LC), has been estimated to be between 10 and 57% (16, 17). This wide range is attributed to inconsistencies in definition and reporting. LC is characterized by many extrapulmonary symptoms that overlap with those commonly seen in HSD, including musculoskeletal pain, dysautonomia disorders with or without associated small fiber neuropathy, cognitive disorders, and fatigue (18). ME/CFS is also often associated with viral infection or reinfection in up to 80% of patients, and symptoms also significantly overlap with those seen in HSD (19). While the exact pathophysiology remains elusive, the major putative etiologies of both LC and ME/CFS include persistent viral remnants, persistent cell-mediated inflammation, endothelial dysfunction, and dysautonomia (16, 20–22). As part of this ongoing inflammatory process common to individuals with LC and ME/CFS, mast cell activation and degranulation may occur, resulting in the initiation of pro-inflammatory cytokine cascades leading to hyperinflammation within the connective tissue of individuals with PASC and ME/CFS without hypermobility (21, 23). These inflammatory changes in connective tissue may lead to further connective tissue damage in patients, thus worsening or inducing hypermobility. Acute infection with SARS-CoV-2 has also been associated with abnormal host immune responses to traumatized connective tissue and elevations in antinuclear antibodies, associated with connective tissue disease in patients with hypermobility (24).

In patients with LC and dysautonomia, there is often a hyperadrenergic state, which drives many of the pathophysiologic mechanisms of hyperadrenergic POTS (25). The severity of POTS is exacerbated when patients have elements of neuropathic POTS, which is often driven by small fiber neuropathy and decreased vascular tone, both of which are present in patients with hEDS/HSD (26–28). Adrenaline, a key effector hormone of the sympathetic nervous system, is elevated in hyperadrenergic POTS. It has been shown to inhibit the function of human fibroblasts and modulate the expression of local growth factors, thereby potentially explaining a mechanism of adrenaline-induced connective tissue dysfunction (29, 30). In patients with persistent exposure to elevated catecholamines and inflammatory processes, HSD may develop or worsen due to these changes. This has been reported in a case series of five women who did not have joint hypermobility before having had COVID but met the criteria for HSD after the development of LC (31). Patients with HSD are also at a 30% higher risk of developing LC and are at a higher risk of having higher fatigue levels with LC (12).

The strong relationship between hypermobility and chronic fatigue with post-exertional malaise is well-described, and there is some evidence connecting these conditions via mitochondrial dysfunction (32–35). Mitochondrial dysfunction may lead to hypermobility secondary to muscle hypotonia, and this may be an important cause of acquired hypermobility as seen in infection-associated chronic illnesses (36). In fact, mitochondrial dysfunction has been definitively shown to be a component of post-exertional malaise in the muscle tissues of patients with LC (37). Further, mitochondrial dysfunction leads to impaired functioning of myofibroblasts, which, in turn, will lead to dysregulated cellular immunity via secretion of cytokines, chemokines, and cellular growth factors, as well as dysregulated enzymatic activity, which directly affects the integrity of the extracellular matrix of the connective tissue (38). Several case reports have outlined mitochondrial genetic mutations associated with Ehlers–Danlos syndrome, in particular hEDS, which may imply a role of mitochondrial function in the maintenance of connective tissue integrity (39, 40).

## Additional putative mechanisms

Of note, in the series of patients who developed HSD after COVID-19 infection, all five patients were identified as having a C677T or A1298C polymorphism in *MTHFR* (methylenetetrahydrofolate reductase gene) and responded to methylfolate and methylcobalamin therapy. This is consistent with previous literature findings that proposed the link between the C677T or A1298C polymorphisms in *MTHFR*, which may occur in up to 35% of the population, and HSD via decreased methylation of the matrix metalloprotease 2 (MMP) gene and increased cleavage of the proteoglycan decorin (41). Increased cleavage of this proteoglycan can lead to an inflammatory cascade that causes significant destruction of the extracellular matrix and, thereby, increased disorganization of the connective tissue and a potential increase in adhesions within the connective tissue, which has been proposed as an etiology of the chronic pain associated with HSD and fibromyalgia (42). MTHFR mutations have also been associated with impaired activity of the mitochondrial respiratory chain, which may drive both hypermobility and fatigue with post-exertional malaise (43). Through their effect on methylation of DNA and thereby variable expression of gene products, MTHFR mutations may be essential in exposomic events such as viral infection leading to infection-associated chronic illnesses (44). In patients with LC and HSD, particularly in female patients with significant multisystem neurologic and musculoskeletal pain symptoms, evaluation for MTHFR polymorphisms should be considered (41, 45).

There is also significant overlap between a diagnosis of neurodivergence and the incidence of hypermobility and fibromyalgia (11, 46). This is supported by two studies wherein family members of patients with both hypermobility and fibromyalgia were evaluated for features compatible with neurodivergence, hypermobility, or fibromyalgia. The incidence of autism spectrum disorder (ASD) or attention-deficit hyperactivity disorder (ADHD) was 39% compared to a prevalence of 2.8% in the relatives of the control group; the incidence of HSD was 30% compared to 8% in the relatives of the control group; and the prevalence of fibromyalgia was 18% compared to 3% in the relatives of the control group (46). Taken together, these findings suggest a common genetic/familial etiology of these three conditions (11). The pathophysiology whereby neurodivergence could contribute to hypermobility and chronic pain is not clear, but it is likely that hypersensitivity to external stimuli and differential responses to pain and stimuli play some role, and patients with neurodivergence have been noted to have dysautonomia with increased basal sympathetic tone and lower parasympathetic tone as well as immune dysregulation with potential contributors of maternal immune activation (47).

## Clinical evaluation and treatment

Screening for HSD should be conducted in patients with complex chronic illnesses (including fibromyalgia, POTS, LC, and ME/CFS), chronic pain conditions (including irritable bowel syndrome and temporomandibular joint disorder), joint pain, and myalgia. The most commonly utilized diagnostic criteria for hEDS/HSD are from the 2017 International Classification of Ehlers–Danlos syndromes, as summarized in Table 1. This screen includes the Beighton criteria for physical examination, and formal measurement requires using a goniometer to accurately assess the degree of hyperextension (3).

**Table 1:** Diagnostic criteria for hEDS.

**Criterion 1**: Generalized joint hypermobility—Beighton score of ≥6 for pre-pubertal children and adolescents, ≥5 for pubertal men and women up to the age of 50 years, and ≥4 for those >50 years of age	
**Criterion 2**: Two or more of the following features (A–C) must be present	
**Feature A**: Systemic manifestations of generalized connective tissue disorder—need ≥5 to be present Unusually soft or velvety skin Mild skin hyperextensibility Unexplained striae such as striae distensae or rubrae at the back, groins, thighs, breasts, and/or abdomen in adolescents, men, or pre-pubertal women without a history of significant gain or loss of body fat or weight Bilateral piezogenic papules of the heel Recurrent or multiple abdominal hernia(s) (e.g., umbilical, inguinal, and crural) Atrophic scarring involving at least two sites and without the formation of truly papyraceous and/or hemosideric scars, as seen in classical EDS Pelvic floor, rectal, and/or uterine prolapse in children, men, or nulliparous women without a history of morbid obesity or other known predisposing medical condition Dental crowding and high or narrow palate Arachnodactyly, as defined in one or more of the following: (i) positive wrist sign (Steinberg sign) on both sides and (ii) positive thumb sign (Walker sign) on both sides Arm span-to-height ≥1.05 Mitral valve prolapse (MVP) mild or greater based on strict echocardiographic criteria Aortic root dilatation with *Z*-score > +2	
**Feature B**: Positive family history, with one or more first-degree relatives independently meeting the current diagnostic criteria for hEDS	
**Feature C**: Musculoskeletal complications—need at least 1 to be present Musculoskeletal pain in two or more limbs, recurring daily for at least 3 months Chronic, widespread pain for ≥3 months Recurrent joint dislocations or frank joint instability, in the absence of trauma^a^	
**Criterion 3**: Exclusion of other conditions. All of these criteria must be met Absence of unusual skin fragility, which should prompt consideration of other types of EDS. Exclusion of other heritable and acquired connective tissue disorders, including autoimmune rheumatologic conditions^b^ Exclusion of alternative diagnoses that may also include joint hypermobility by means of hypotonia and/or connective tissue laxity.	

a Three or more atraumatic dislocations in the same joint or two or more atraumatic dislocations in two different joints occurring at different times, or medical confirmation of joint instability at two or more sites not related to trauma.

b In patients with an acquired connective tissue disorder (e.g., lupus and rheumatoid arthritis), additional diagnosis of hEDS requires meeting both Features A and B of Criterion 2. Feature C of Criterion 2 (chronic pain and/or instability) cannot be counted toward a diagnosis of hEDS in this situation.

Once a diagnosis of hEDS or HSD has been confirmed, patients should be referred to specialized physical and occupational therapy aimed at strengthening the muscles surrounding the joints to reduce the risk of recurrent dislocations, along with training to avoid joint hyperextension (48, 49). Patients with HSD have been noted to have decreased acuity of proprioception and increased latency of muscle activation, which may be related to gait abnormalities, recurrent joint injuries, and musculoskeletal pain (50, 51). Physical therapy interventions have been shown to significantly improve quality of life, functional exercise capacity, and proprioception; therapeutic exercise and motor function testing were the most efficacious interventions. Hydrotherapy, which involves physical therapy exercises in a heated pool, has been recommended as a useful adjunct, as the warmth can help relax muscles and the water provides additional support and some dynamic resistance (52). The ongoing focus of physical therapy is to teach patients how to protect their joints during daily activities through proper body mechanics and strengthening in order to avoid hyperextension and associated injury or strain.

Care should be taken for patients who have fatigue with post-exertional malaise, as exercise can have deleterious effects on these individuals (37). Though there are severe exercise limitations, including resting tachycardia in patients with LC, we recommend that they be managed with pacing as recommended in ME/CFS treatment guidelines (53, 54).

Patients should be referred to a multidisciplinary clinic, and many of the treatments that provide benefits for people with hEDS or HSD are non-pharmacological, including massage, yoga, chiropractic manipulation, and meditation (55). Additionally, multidisciplinary treatment modalities that affect various spheres of health including sleep hygiene, emotional support from friends and family, energy conservation, and coping strategies are all likely to be beneficial to patients.

Pharmacologic treatments may certainly be indicated, but these are symptom-focused and geared to the clinical phenotype with some evidence supporting the role of tricyclic agents such as amitriptyline and non-steroidal anti-inflammatory agents (NSAIDs) such as naproxen for pain in people with hEDS/HSD (56). It should be emphasized that there is no therapeutic role for opioid therapy in patients with hEDS/HSD, and in fact, opioid therapy often worsens pain outcomes (57).

Given the significant overlap between HSD and other complex chronic medical conditions, such as POTS, LC, fibromyalgia, and ME/CFS, therapeutic agents may be trialed as per society recommendations (56). These include β-blockers such as propranolol, mineralocorticoids such as fludrocortisone, and α-agonists such as midodrine in POTS (58–60). Additionally, low-dose naltrexone may be considered for fibromyalgia and ME/CFS (61, 62).

## Discussion

Hypermobile spectrum disorders and hypermobile Ehlers–Danlos syndrome are uncommon conditions occurring in an estimated 10–20% of the population but are enriched in populations with neurodivergence and complex chronic conditions including infection-associated chronic illnesses. There is considerable heterogeneity in clinical presentation, and the etiology of these conditions is currently not fully understood. There are however several potential mechanisms that may have multidirectional interplay in the genesis of hypermobility and its associated conditions. Hypermobility conditions are associated with chronic cell-mediated inflammation, resulting in a pro-inflammatory cytokine/chemokine environment in the cellular matrix, and this is often influenced by external conditions including neurodivergence and exposure to environmental triggers such as viruses, which then induce exposomic change. Disease states that may impact methylation such as MTHFR mutations may make these exposomic changes more likely and thus play a role in the generation of hypermobility. It is interesting to consider that while hypermobility may predispose individuals to infection-associated chronic illnesses and the chronic inflammation that is associated with this state; similarly, infection-associated chronic illnesses may induce hypermobility through a combination of connective tissue inflammation and destruction. Given the large numbers of people affected by long COVID, it is critical to screen for hypermobility and other related conditions and enroll patients into multidisciplinary treatment teams to provide individualized treatment plans to maximize function and minimize symptoms.

## Data Availability

The original contributions presented in the study are included in the article/supplementary material, further inquiries can be directed to the corresponding author.
